# Supplementary material for: Prevalence of advanced hepatic fibrosis and individualization of associated risk factors by Bayesian analysis in MASLD patients in French cardio-metabolic health networks
Source: PLoS One. 2025 Jan 30;20(1):e0316158. doi: 10.1371/journal.pone.0316158 (PMC11781707; doi:10.1371/journal.pone.0316158)
Supplement: Attachment — Submitted filename: Response to reviewers vf.docx [file pone.0316158.s001.docx]

Strasbourg, november, 2024

Dear Editor,

Thank you for your fine comments.

Please find by there the Response to your comments and to the reviewvers.

Yours sincerely,

Frédéric Chaffraix

**Response to Editor**

- The manuscript has been reviewed according to the guidelines for authors.
- The term « Bayesian analysis »,which contributes to the originality of the article has been added to the title.
- Unfortunately, we are unable to publish the study data due to French regulatory constraints. In order to publish the data, we would have to modify it beforehand to meet the expectations of the ethics committees. If this were the case, readers would not be able to retrieve the results of the study from the data. However, if you wish to explore specific data in the database, we would be happy to respond to your requests.
- Among the limitations of the study the declarative nature of alcohol and tobacco consumption has been added.
- Among the references, reference 1 has been replaced by a more recent reference and reference 42 has been added.

**Response to reviewers’ comments**

Reviewer #1 :

Accessibility to FibroScan in cardio-metabolic health networks is conditioned on the intervention of a mobile team with the necessary equipment. As part of a sequential approach the dissemination of FIB-4, which is the most widely established and available blood test, should make it possible to restrict the number of FibroScan and to promote their accessibility. In order to guarantee the latter a network organization at the level of a health territory with a pooling of personnel and FibroScan would be justified with coordination by reference centers. For the record, the design of our study was developed prior to the availability of diagnostic algorithms for using noninvasive tests for risk stratification of patients with suspect NAFLD (MASLD) in clinical practice.

Reviewer #2

The alcohol consumption thresholds used in our study are consistent with those used in the recent classification of different categories of steatotic liver disease (see reference 1). They allow to eliminate a possible MetALD (MASLD with moderate alcohol intake, 30-60g/day in men and 20-50g/day in women).

Indeed, alcohol consumption was more common among men than among women (132/194 =68% vs 137/383 = 36%), but it did not increase the risk of advanced fibrosis in the Bayesian logistic regression (see Table 3). For information, no effect of past regular alcohol consumption was observed for all variables studied as shown in the following table.
